# Modification of polymethylmethacrylate bone cement with halloysite clay nanotubes

**DOI:** 10.1186/s12903-024-04600-3

**Published:** 2024-08-04

**Authors:** Tamer M. Hamdy

**Affiliations:** Restorative and Dental Materials Department, Oral and Dental Research Institute, National Research Centre (NRC), Giza, Dokki, 12622 Egypt

**Keywords:** Bone cement, Polymethylmethacrylate, PMMA, Halloysite, Clay, Nanotubes, Compressive strength, Flexural strength

## Abstract

**Background:**

Polymethylmethacrylate (PMMA) bone cement is used in orthopedics and dentistry to get primary fixation to bone but doesn’t provide a mechanically and biologically stable bone interface. Therefore, there was a great demand to improve the properties of the PMMA bone cement to reduce its clinical usage limitations and enhance its success rate. Recent studies demonstrated that the addition of halloysite nanotubes (HNTs) to a polymeric-based material can improve its mechanical and thermal characteristics.

**Objectives:**

The purpose of the study is to assess the compressive strength, flexural strength, maximum temperature, and setting time of traditional PMMA bone cements that have been manually blended with 7 wt% HNT fillers.

**Methods:**

PMMA powder and monomer liquid were combined to create the control group, the reinforced group was made by mixing the PMMA powder with 7 wt% HNT fillers before liquid mixing. Chemical characterization of the HNT fillers was employed by X-ray fluorescence (XRF). The morphological examination of the cements was done using a scanning electron microscope (SEM). Analytical measurements were made for the compressive strength, flexural strength, maximum temperature, and setting time. Utilizing independent sample t-tests, the data was statistically assessed to compare mean values (*p* < 0.05).

**Results:**

The findings demonstrated that the novel reinforced PMMA-based bone cement with 7 wt% HNT fillers showed higher mean compressive strength values (93 MPa) and higher flexural strength (72 MPa). and lower maximum temperature values (34.8 °C) than the conventional PMMA bone cement control group, which was (76 MPa), (51 MPa), and (40 °C), respectively (*P* < 0.05). While there was no significant difference in the setting time between the control and the modified groups.

**Conclusion:**

The novel PMMA-based bone cement with the addition of 7 wt% HNTs can effectively be used in orthopedic and dental applications, as they have the potential to enhance the compressive and flexural strength and reduce the maximum temperatures.

## Background

Polymers are essential to various aspects of dentistry, including restorative, rejuvenating, and preventative care [1, 2]. In the field of dentistry, bone cements are utilized for anchoring dental implants in specific situations, retrograde filling, sinus floor augmentation, and bone defect filling [3]. Polymethyl methacrylate (PMMA) and calcium phosphate cements (CPC) are the two most widely employed bone cements [4]. Till now, PMMA-based bone cements have been considered the gold standard for anchoring artificial prosthetic appliances to the bone structure [5]. These two components polymerize once liquid and powder phases are combined, forming a reactive malleable paste [6]. After six minutes of mixing, the temperature of the cement increased due to the release of heat that occurs during the exothermic polymerization process, which may cause tissue necrosis [7]. Therefore, the applications of PMMA-based bone cements are inadequate in cases that require a large volume of materials to be used [8].

Transferring weight from the prosthesis to the bone is the primary purpose of the bone cement. Therefore, the mechanical characteristics of bone cement have been employed to evaluate its efficacy [9]. Compressive and flexural strength tests are the two primary mechanical tests used to evaluate the bone cement as described by ISO and ASTM guidelines [10, 11]. For filling cavities, bone cement should have a minimum compressive strength of 30 MPa, which is comparable to naturally occurring spongy bone. While the minimum compressive strength should be 70 MPa when used for the cementation of prosthetic appliances [12].

PMMA-based bone cements are primarily being advanced through changes that enhance their mechanical and biological properties. Previous studies have been designed to improve the properties of the PMMA-based bone cements, including the incorporation of metals such as silver, gold, and titanium nanoparticles into the polymeric matrix to induce antimicrobial effects [13]. Moreover, Bioactive glass (BG), hydroxyapatite (HA), and tricalcium silicate nanofillers were used as strengthening agents [3, 4, 14].

Composites made of organic polymers and inorganic materials are very desirable because they have unique structural and biochemical characteristics that are comparable to those of naturally occurring hybrid systems like bones [15]. Halloysite clay nanotubes are a clay mineral, chemically it considered as a subclass of kaolin clays [16]. HNTs are a naturally occurring inorganic biomaterials. Chemically it is aluminosilicate nanotube, with chemical formula (Al_2_Si_2_O_5_(OH)_4_.2H_2_O). Their porous dual-layer structure and composition of Al, Si, H, and O encourage their application as reinforcement nanofillers in various fields [17].

Recent studies demonstrated that the addition of HNTs to a polymer can enhance its mechanical and thermal characteristics even at low concentrations of 3–7 wt% [18, 19]. HNTs have been identified as one of the safest inorganic nanomaterials to be employed in variety of applications even at quite high concentrations (up to 0.5 mg/mL) as it is easily to be eliminated by the action of macrophages [20].

The PMMA-based bone cements have several drawbacks, such as low mechanical properties, heat generation, and a lack of bioactivity. As a result, several additions could be employed to improve the efficiency of the bone treatment. The aim of this research is to modify the PMMA-based bone cements with halloysite clay nanotubes to improve their mechanical properties and decrease their heat generation. The null hypothesis was that the reinforcement of the traditional PMMA-based bone cement by addition of 7 wt% halloysite clay nanotubes would improve the compressive strength, flexural strength, and reduce maximum temperature, with no effect on setting time compared to the unmodified control group.

## Methods

A commercially available PMMA-based bone cement (Cemex Isoplastic, Tecres S.P.A., Sommacampagna, Italy) was used. Commercial Halloysite Clay Nanotubes powder (empirical formula Al_2_Si_2_O_5_(OH)_4_.2 H_2_O) was also used (Nano Research Elements, New Delhi, India). The specifications of the HNTs powder; color was yellow/off-white; purity > 99.9%; average particle size (APS) > 80 nm. The commercial materials used in the preset study were listed in Table 1.

**Table 1:** The commercial materials used in the present study

Materials	Manufacturer	Batch no.
Cemex Isoplastic Powder (75 wt% of Cement): Polymethyl methacrylate: 84.30 wt%; Barium sulfate: 13.00 wt%; Benzoyl peroxide: 2.70 wt%. Liquid (25 wt% of Cement): Methyl methacrylate: 99.10 wt%; N-N-dimetylo-p-toluidyne: 0.90 wt%; Hydroquinone: 75 ppm wt%	Tecres S.P.A., Sommacampagna, Italy	AA6596
Halloysite Clay Nanotubes powder: Al_2_Si_2_O_5_(OH)_4_.2 H_2_O	Nano Research Elements, New Delhi, India	06FESPK4563R1ZF

### Characterization of used HNTs fillers

Chemical characterization was conducted by a non-destructive X-ray fluorescence (XRF) analysis (X-MET3000TXR, Oxford Instruments GmbH Co., Borsigstrasse, Germany) was used for the chemical characterization of used HNTs fillers in order to confirm their chemical composition [21]. In addition, morphological characterization of the prepared bone cements after specimen setting was done using a scanning electron microscope (SEM) (Quanta 250 FEG, FEI Company, Hillsboro, OR, USA). It was performed at 1000X magnification and an accelerating voltage range of 20.0 kV.

### Sample size calculation

The sample size calculation was based on a similar study [22, 23], with an alpha level of 0.05 and a power of 85%, a sample size was determined using G*Power software version 3.1.9.7 (Heinrich Heine University Duesseldorf, Duesseldorf, Germany). The minimum sample size needed with this effect size is *n* = 10 per group to test compressive strength, flexural strength, and temperature changes.

### Study design

**Fig. 1:**
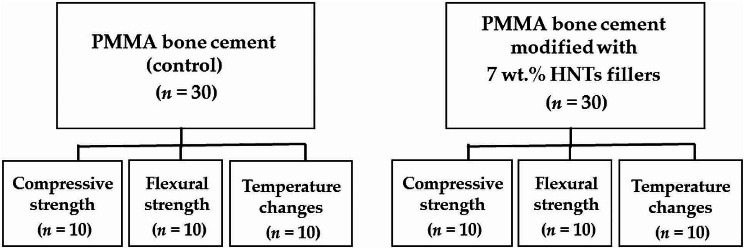
A schematic illustration of the groups

A total of 60 specimens were used. The specimens were divided into two main groups according to each type of bone cements (*n* = 30), they were divided into three subgroups of specimens (*n* = 10) to examine compressive strength, flexural strength, and temperature changes (maximum temperature and setting time), as represented in Fig. 1.

### Preparation of the specimens

The control group was prepared by mixing the conventional commercially available PMMA-based bone cement powder with their liquid; the modified group was obtained by hand-mixing of 7 wt% Halloysite Clay Nanotubes fillers with the conventional PMMA-based bone cement powder, and then mixing with their liquid. Manual mixing of powder and liquid in accordance with manufacturer’s instructions was done in a bowel for 30 s. After mixing, the cement dough from each group was poured into a custom-made special Teflon molds according to each test. A polyester strip was placed to prevent air trapping, a glass slide was used to gently compress the materials on both sides of the mold. After the manufacturer’s recommended setting time, specimens were removed out of the mold. Next, the specimens inspected for any defects, then polished with silicon carbide papers 2000 grit, then visually re-inspected for any defected specimens.

### Compressive strength measurement

Ten cylinder specimens per group (diameter = 6 mm, height = 12 mm) were prepared according to ASTM F451 − 21 Standard Specification for acrylic bone cement (2021) [10]. The complete set specimens were removed from the molds and stored in an incubator (CBM, S.r.l. Medical Equipment, 2431/V, Cremona, Italy) for 24 ± 2 h in 50 ± 10% relative humidity at 23 ± 2 °C. Specimens were loaded, the compression was measured at a 20 mm/min speed in a universal testing machine (Shimadzu Autograph AG-X Plus, Kyoto, Japan) until failure under room temperature [10].

### Flexural strength measurement

A flexural strength was evaluated using 4-point bending according to the ISO 5833-02 [23]. Specimens of rectangular specimens of 75 mm × 10 mm × 3 mm were prepared [23]. The specimens were examined by a universal testing machine (Model 3345; Instron Industrial Products, Norwood, MA, USA). The load was applied to the center of the specimens, with a crosshead speed of 5 mm/min, the specimens were loaded till fracture. Bending strengths were computed. Bending strength were calculated according to the ISO 5833-02 [23].

### Temperature changes measurement

Cylindrical specimens (*n* = 10 per group) measuring 6 mm diameter, and 12 mm height were prepared. During the setting process, the temperature change in the center of each specimens was measured using a digital infrared radiation thermometer (SK-890, Sato Keiryoki MFG. Co., Tokyo, Japan). The temperature changes was calculated in according to ISO 5833:2002 for acrylic bone cement [23], the setting time is defined as the time point determined from the start of mixing until the average of the maximum temperature and ambient temperature (23ºC). The maximum temperature (polymerization temperatures) was calculated from the graph.

## Statistical analysis

The Statistical Package for the Social Sciences (SPSS) 16.0 statistical program (IBM-SPSS version 27.0, New York, NY, USA) was used to conduct the statistical study. Shapiro-Wilk tests revealed that the data had a normal distribution. The mean compressive strength (MPa), flexural strength (MPa), maximum temperature (°C) and setting time (min.) for the PMMA Bone Cement (control) and PMMA Bone Cement + 10 wt% HNTs were compared using an independent sample T test. A significant threshold of *P* < 0.05 was established.

## Results

### Chemical characterization results

Table 2 displays the chemical composition of the employed HNT fillers that were subjected to XRF analysis. The composition included halloysite Al_2_Si_2_O_5_(OH)_4_.2H_2_O, as the results showed.

**Table 2:** Chemical compositions of the HNT fillings (mass%) determined by XRF analysis

Compositions	Mass%
Si	21.71
Al	20.90
O	41.74
Fe	13.70
Ca	0.63
K	0.69
Na	0.53
Ti	0.10

### The SEM morphological results

**Fig. 2:**
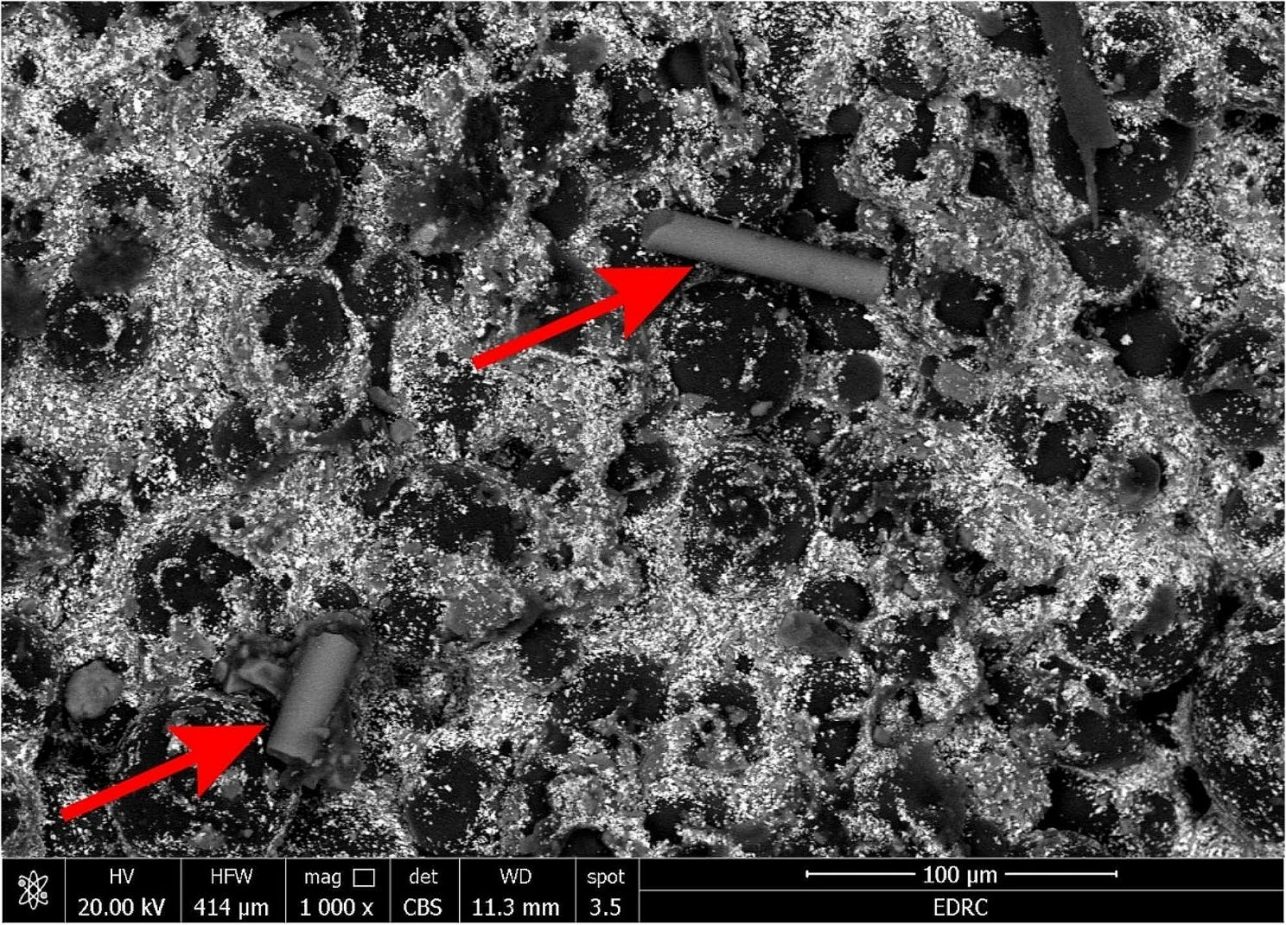
A representative SEM image of the modified group, the arrow refers to the HNT filler

The SEM analysis of the bone cement specimens after setting was examined at a magnification of 1000 X, as represented in Fig. 2. The SEM micrograph showed a homogeneous, short, and straight tubular structure of the HNT filler embedded within polymeric matrix with a uniform distribution.

### Analytic test results

#### Results for compressive strength

Table 3 displays the results of the mean compressive strength values. The modified PMMA bone cement group including 7 wt% HNTs had a significantly greater compressive strength value (93 MPa) compared to the control PMMA bone cement group (76 MPa), (*P* = 0.0001*).

**Table 3:** The mean and standard deviation values of compressive strength for the two groups

Test	PMMA Bone Cement (control)	PMMA bone cement + 7 wt% HNTs	*P* value
Compressive strength (MPa)	76 ^a^±1.9	93 ^b^±1.2	0.0001*

* denotes a significant difference for distinct small letters in the same row when *P* < 0.05

### Results for flexural strength

Table 4 displays the findings of the flexural strength values. The modified PMMA bone cement group containing 7 wt% HNTs had a significantly greater flexural strength value of (51 MPa) compared to the control PMMA bone cement group (32 MPa), (*P* = 0.0001*).

**Table 4:** The mean and standard deviation values of flexural strength for the two groups

Test	PMMA Bone Cement (control)	PMMA bone cement + 7 wt% HNTs	*P* value
Flexural strength (MPa)	51 ^a^±1.2	72 ^b^±1	0.0001*

* denotes a significant difference for distinct small letters in the same row when *P* < 0.05

### Results for temperature changes

**Fig. 3:**
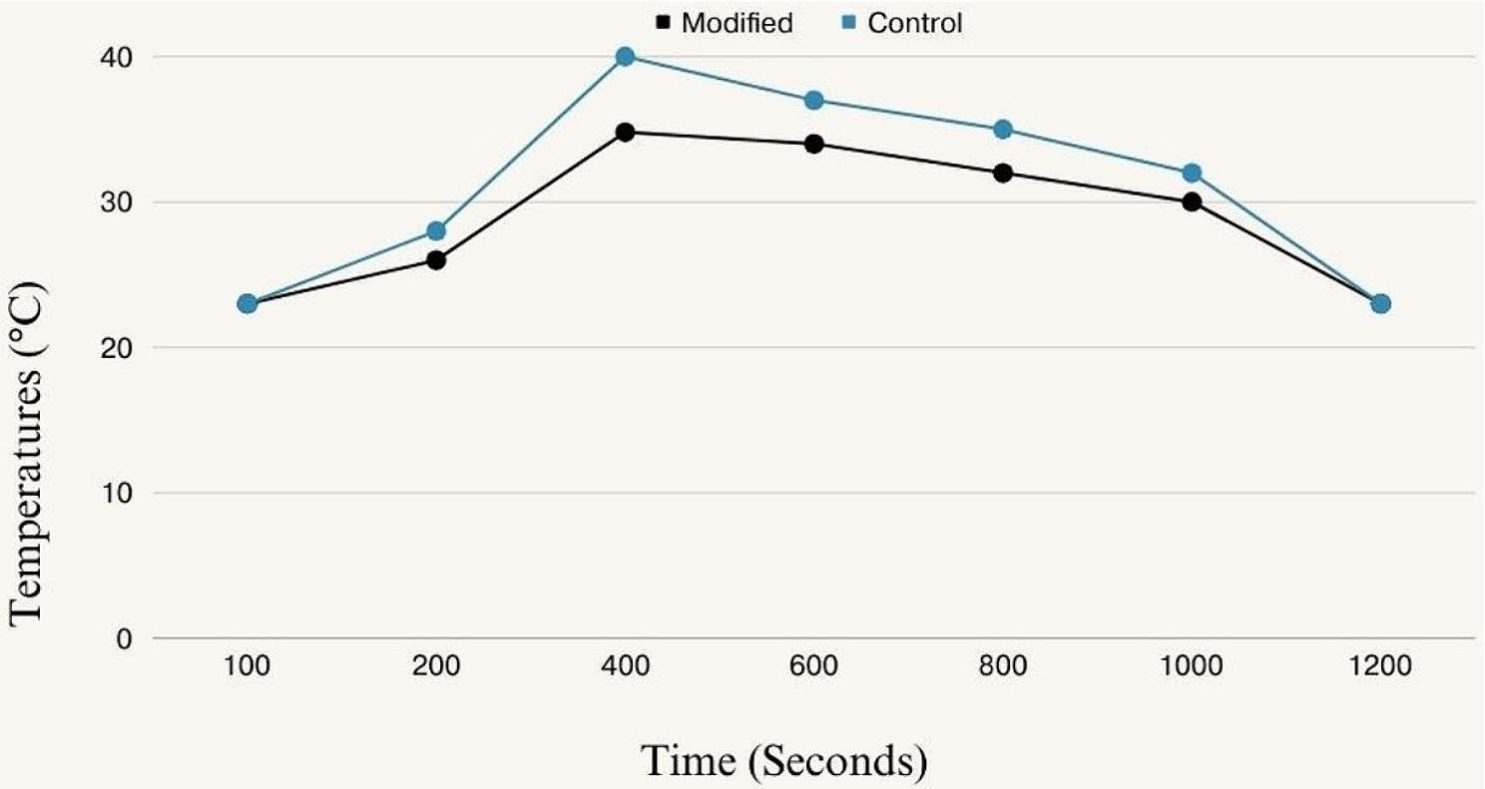
Line chart represent the temperature changes of the tested bone cement groups over time

The Maximum temperature (T max) results was presented in Table 5. There was a significant difference between the two tested groups in their maximum temperature. The modified PMMA Bone Cement with 7 wt% HNTs showed significantly lower maximum temperature compared to the control PMMA bone cement. The control groups exhibited a maximum temperature of 40 ± 0.7 °C, peak temperature was observed 400 s after the solid and liquid components were mixed. In addition, the modified groups showed a maximum temperature of 34.8 ± 0.1 °C, with a peak temperature recorded at 400 s (*P* = 0.0001*). Moreover, the mean value of setting time was represented in Table 6. There was no significant difference between the setting time of the two tested groups (*P* > 0.05). The temperature changes of specimens over time are shown in Fig. 3.

**Table 5:** The maximum temperature mean and standard deviation values between the two groups

Test	PMMA Bone Cement (control)	PMMA bone cement + 7 wt% HNTs		*P* value
Maximum temperature (T max) (°C)	40 ^b^±0.7		34.8 ^a^±0.1	0.0001*

* denotes a significant difference for distinct small letters in the same row when *P* < 0.05

**Table 6:** The setting time mean and standard deviation values between the two groups

Test	PMMA Bone Cement (control)	PMMA bone cement + 7 wt% HNTs		*P* value
Setting time (min.)	13.1 ± 0.1		13.3 ± 0.2	0.1

## Discussion

The utilization of bone substitutes and cements in dentistry has significantly expanded recently due to the demand to repair bone defects [24]. PMMA is most frequently employed polymer in dentistry is PMMA [25]. PMMA-based bone cements exhibit high biocompatibility with human tissues [26]. However, it has inferior mechanical performance, which in turn may cause the cement to become loose or dislodged and cause the formation of an unstable interface with bone [26, 27]. Table 7 provide the average values of some mechanical properties of PMMA and PMMA-based bone cement [25, 28, 29].

**Table 7:** Comparison between some mechanical properties of PMMA and PMMA-based bone cement

Mechanical properties	PMMA	PMMA-based bone cement
Compressive strength	76 MPa	78 MPa
Flexural strength	90 MPa	57 MPa
Tensile strength	48–62 MPa	30 MPa
Fracture toughness	1.86 MPa.m^1/2^	1.6–2.1 MPa.m^1/2^

The PMMA cement displays exothermic behavior during the polymerization of MMA; the generated heat may cause damage and necrosis to the surrounding bone or other tissues adjacent to the implant [22, 30]. All these limitations may lead to the failure of the bone cement. Considerable work has been put into enhancing the mechanical properties and reducing heat generation in order to address these limitations and expand their clinical performance and durability [31].

The HNT has two layers of aluminosilicate, which provides high mechanical strength, thermal stability, and biocompatibility [32]. Combining HNTs with polymers could result in effective nanocomposites with enhanced reinforcing effects [33]. The concentration of HNT fillers was adjusted to 7 wt%, as it is established that they provide the greatest reinforcing effects when incorporated into polymeric composites [18, 19].

The XRF data is a successful methods for determining the chemistry of the minerals [34]. The acquired results of the XRF confirms the successful formation of HNT [35]. The XRF spectra shows the presence of Al, and Si, which form the main composition of SiO_2_ and Al_2_O_3_ of Halloysite [34]. Moreover, the SEM micrograph revealed a homogenous distribution of the HNT filler within the polymeric matrix, which is an important factor for the reinforcing effect [25].

The purpose of the current study is to assess the impact of adding 7 wt% HNT fillers to PMMA-based bone cement that is commercially available on compressive strength, flexural strength, and maximum temperatures. The null hypothesis, which stated that adding 7 wt% HNT to conventional PMMA-based bone cement would improve the compressive strength, flexural strength, and reduce heat generation compared to the unmodified control group, was accepted as the modified groups showed a higher compressive strength (93 MPa), a higher flexural strength (72 MPa), and lower maximum temperature values (34.8 °C) than the conventional control group with no effect on the setting time. The minimum requirement for the PMMA-based bone cements adopted by the ISO 5833:2002 standard specifications was (70 MPa) for the compressive strength and (50 MPa) for the bending (flexural) strength [10]; both the commercially available PMMA-based bone cement and the modified groups meet this requirement. As regards the compressive strength, the modified groups possessed a significantly higher compressive strength. The proposed reinforcing effects of HNT fillers could be the explanation for this finding [32]. It was established that the addition of approximately 3–5 wt% of HNTs to dental-based cement can significantly improve their mechanical properties such as compressive, tensile, and flexural strength [36]. Furthermore, one possible explanation for the reinforcing action of HNT could be their high stiffness and high sensitivity to dispersion in the polymer matrix [37]. In addition, HNTs are made up of many siloxane groups and a few hydroxyl groups, which provide the material the capacity to form hydrogen bonds and, thus, a higher dispersion potential [38].

Our finding comes in accordance with the results obtained by Salaman et al. [39]. They found that the addition of halloysite to the polyurethane provides nanocomposites with enhanced compressive strength. Additionally, the research done by Ravichandran et al. [40] showed that epoxy resin with a homogeneous dispersion of 3% weight HNTs has higher compressive strength than other nanocomposites without HNT fillers.

Furthermore, the flexural strength results of the modified groups also showed higher performance than the control groups, which may be caused by the action of HNT incorporation, which acts as a reinforcing phase that increases the resistance to brittle cracking and flexural strength [12, 32]. These findings are consistent with research by Barot et al. [41], which found that adding HNT fillers to resin composites up to 7% improved their compressive and flexural strengths in comparison to control. While degradation in the mechanical properties was showed by increasing the HNT fillers to 7.5% and 10 wt%. This could be the result of the HNT agglomeration resulting in a loss of mechanical qualities. Moreover, a previous studies that conducted by Franciszczak et al. [32] showed that addition of halloysite nanotube to polypropylene enhance their flexural strength. Furthermore, when Rojas et al. [23]. incorporated 0.3 wt% graphene oxide into PMMA bone cement, they found that the compressive strength and modulus of compression increased in the modified groups compared to the unmodified groups.

During setting, PMMA cement releases heat that can affect the surrounding healthy cells and may cause their damage. Therefore, the cement setting behavior is crucial since it may be regulated by adding components to PMMA cement such as bioactive glass and other ceramic-based biomaterials [42].

The maximum temperature produced by the plain PMMA (control) groups is about 40 °C. In the PMMA system, MMA polymerization is considered highly exothermic. The amount of heat produced by the liquid-phase polymerization reaction is equivalent to the peak temperature reached, which in living tissues could result in necrosis [22]. Nevertheless, the maximum temperature during the polymerization process was dropped; as the proportion of HNT fillers in PMMA bone cement was adjusted to 7 wt%, the maximum temperature was reduced to 34.8 °C. These findings may be explained by the insulated ceramic features of the incorporated HNT, which may slow the heat transfer release during the polymerization procedure compared to the control groups [22, 43]. Therefore, during the cement’s setting reaction, the modified group would be far safer for the nearby tissues. As regard the setting time, no significant difference was noticed among the groups, this may attribute to the lower concentration of HNT filler (7 wt%) that not significantly affect the polymerization process. Moreover, it was determined that all the obtained results of the control and modified bone cements fall within the range of the ISO 5833:2002 requirements for acrylic bone cement [23], which permit a maximum temperature of up to 90 °C and a setting time ranging from 3 to 15 min.

Wei et al. [22] revealed that the addition of 30 wt% tricalcium silicate to PMMA bone cement reduced the maximum temperature without significantly affecting the mechanical and handling characteristics of the composite cements. They attribute the decrease in exothermic reaction to the insulated ceramic capacity of the tricalcium silicate filler, which may impede heat transfer during PMMA polymerization. Furthermore, Miola et al. [14], investigated the addition of 10 wt% silver-doped bioactive glass particles to PMMA-based bone cement. They found that the maximum temperature in the control group was 58 °C, while in the modified group it decreased to 51 °C. Moreover, the setting time was 8.3 min in the control group and increased to 9.3 min in the modified group. They estimated that the ceramic-based filler reduces the exothermic reaction while setting time by increasing the amount of filler due to slowing the polymerization reaction.

Despite the research conducted in preclinical models, additional clinical assessments are necessary to determine the precise effects of HNT on the human body in order to provide a reliable and comprehensive clinical treatment in the future. Lack of salinization between fillers and polymeric matrix is considered a limitation of the study; in addition, the study was conducted only using one filler concentration. Therefore, it is recommended that future studies investigate the effects of including HNT at higher concentrations in PMMA-based bone cement to identify the optimal concentrations required to improve the mechanical characteristics. Moreover, it is recommended to investigate the cement viscosity and bond strength. In addition, further in vivo examination of the cell proliferation, regenerative potential, and bioactivity of the modified cement is required to ensure the probability of biomedical applications.

## Conclusions

The addition of 7 wt% HNT to the PMMA-based bone cement could enhance the compressive and flexural strength and reduce the maximum exothermic temperature observed during the cement polymerization procedure. Thus, the novel PMMA-based composite cement with the addition of HNT in the selected concentration could potentially be applied in dentistry and orthopedic applications as a new bone cement.

## Data Availability

The data that support the findings of this study are available from the corresponding author upon reasonable request.

## Abbreviations

PMMA: Polymethylmethacrylate
HNTs: Halloysite nanotubes
CPC: Calcium phosphate cements
ISO: International organization for standardization
ASTM: American society for testing and materials
MPa: Megapascal
XRF: X-ray fluorescence
BG: Bioactive glass
HA: Hydroxyapatite
wt.%: Weight%
mg: Milligram
mL: Milliliter
SPSS: Statistical package for the social sciences
